# Quantifying kinematic differences between land and water during squats, split squats, and single-leg squats in a healthy population

**DOI:** 10.1371/journal.pone.0182320

**Published:** 2017-08-02

**Authors:** Anna C. Severin, Brendan J. Burkett, Mark R. McKean, Aaron N. Wiegand, Mark G. L. Sayers

**Affiliations:** 1 School of Health and Sports Sciences, University of the Sunshine Coast, Sippy Downs, Queensland, Australia; 2 School of Science and Engineering, University of the Sunshine Coast, Sippy Downs, Queensland, Australia; Nanyang Technological University, SINGAPORE

## Abstract

Aquatic exercises can be used in clinical and sporting disciplines for both rehabilitation and sports training. However, there is limited knowledge on the influence of water immersion on the kinematics of exercises commonly used in rehabilitation and fitness programs. The aim of this study was to use inertial sensors to quantify differences in kinematics and movement variability of bodyweight squats, split squats, and single-leg squats performed on dry land and whilst immersed to the level of the greater trochanter. During two separate testing sessions, 25 active healthy university students (22.3±2.9 yr.) performed ten repetitions of each exercise, whilst tri-axial inertial sensors (100 Hz) recorded their trunk and lower body kinematics. Repeated-measures statistics tested for differences in segment orientation and speed, movement variability, and waveform patterns between environments, while coefficient of variance was used to assess differences in movement variability. Between-environment differences in segment orientation and speed were portrayed by plotting the mean difference ±95% confidence intervals (CI) throughout the tasks. The results showed that the depth of the squat and split squat were unaffected by the changed environment while water immersion allowed for a deeper single leg squat. The different environments had significant effects on the sagittal plane orientations and speeds for all segments. Water immersion increased the degree of movement variability of the segments in all exercises, except for the shank in the frontal plane, which showed more variability on land. Without compromising movement depth, the aquatic environment induces more upright trunk and shank postures during squats and split squats. The aquatic environment allows for increased squat depth during the single-leg squat, and increased shank motions in the frontal plane. Our observations therefore support the use of water-based squat tasks for rehabilitation as they appear to improve the technique without compromising movement depth.

## Introduction

The benefits of aquatic-based exercise constitute its common practice in rehabilitation, recovery, and fitness [1–3]. The reduced joint loading and external resistance provided by the buoyancy and viscosity are easily modifiable by manipulation of immersion depth [4], and practitioners can adjust the degree of offloading and resistance to progress exercises in a safe manner. Further, researchers suggests that viscosity slows movements and therefore prolongs the time an individual has to regain postural control and that buoyancy provide additional support [5, 6], which suggest that balance in the aquatic environment also is likely affected by water depth [7, 8]. The adaptability of aquatic exercise protocols means that they are suitable for exercise and rehabilitation of individuals with injury and pathology, as well as older and obese populations, where full gravitational loading might be inappropriate [4]. Previous research has indicated differences in muscle activity, joint angles, and movement speeds when walking and running in water [9, 10] and during isolated knee flexion-extension tasks [11], but the influence of the aquatic environment on closed-chain exercises often prescribed for rehabilitation and fitness programs has not been well researched. This has left practitioners without a comprehensive understanding of kinematic implications of water immersion on prescribed exercises, which potentially reduces their ability to ensure optimal efficacy of water-based exercise.

The squat exercise (and its variants) is common to numerous aquatic and land-based rehabilitation programs, with these movements described as functional, closed-chain exercises that involve all major muscles and joints of the lower body [12, 13]. In addition to the traditional squat, research supports the prescription of the split squat (SS) and single-leg squat (SLS), and their land-based kinematics are well documented in the literature [13, 14]. It is likely that squat kinematics differs, as is the case with walking and running [9, 10], when performed in water rather than on land. However, despite the significant body of literature on the land-based kinematics of these exercises, their water-based kinematics are not well investigated. To provide practitioners with the understanding to ensure optimal application of aquatic exercises, further examination of how the two environments affect squat kinematics is needed.

The aquatic environment is often considered a safer exercise setting than land, based on the fact that the density of the water slows movement speeds [5, 15], and thereby has been suggested to improve control and stability of movements [9]. Further, researchers have shown that slower squatting speeds reduce shear and compressive joint loads on the spine and knees [16, 17]. Additionally, it is important to control and monitor movement speeds during the ascending and descending phases of an exercise as they induce different muscular responses [18, 19], and biomechanics [20, 21]. In water, the buoyancy force decreases the loading during the descending phase, and during the ascending phase, it provides lifting assistance [22], while the viscosity also provides additional resistance [23]. Accordingly, water immersion likely affects the kinematics of the phases differently, although at the time of submission this has not been reported in the scientific literature, and increased understanding of these implications would be useful practitioners when employing the aquatic environment.

Water-based exercise creates numerous eddies, waves, and currents around the body, causing the accompanying forces to change constantly in response to body movements and water depth. According to the concepts described in dynamical systems theory [24], exercising immersed in this ever-changing environment will require the body to adapt and thus increase its movement variability. While research in this area is still relatively new, exercises that increase movement variability are probably beneficial as reduced variability linked to overuse injuries [25]. Further, as injured populations commonly portray reduced adaptability [24], increased movement variability is likely advantageous for rehabilitation programs. However, despite research on movement variability have espoused its roles in athletic performance and rehabilitation, its impact in the aquatic environment has received little attention and remains largely unreported.

The growing use of aquatic exercises in training and rehabilitation, coupled with the relative absence of objective research on the influence of the aquatic environment on movement patterns for squats and squat based exercises were the key motivations for this study. Accordingly, this study aims to (1) quantify differences in segmental orientation and speed between land- and water-based squats, SS and SLS during the ascending and descending phases, and (2) examine whether the aquatic environment affects the degree of movement variability of the exercises when performed by individuals without previous exposure to water-based squats. It was hypothesized that water immersion would change the segmental orientations and reduce the speeds compared to land, and that the degree of movement variability would increase due to unfamiliar environmental constraints.

## Materials and methods

### Participants

Twenty-five healthy university students (11 females: 1.64±0.06 m, 59.2±10.3 kg, 21.6±2.3 yrs., and 14 males: 1.77±0.08 m, 75.3±10.5 kg, 22.6±3.3 yrs.) volunteered for participation in this study. The participants were healthy at the time of testing and had at least 3 years’ experience in gym-based activity with no prior exposure to aquatic-based exercise. Inclusion criteria ensured that participants were without any past lower limb surgeries and injury free at the time of testing. Self-reported leg dominance was recorded (left = 2, right = 22) by determining participants preferred kicking leg, and written informed consent was obtained prior to any testing in accordance with the approval from the University of the Sunshine Coast Human Research Ethics Committee.

### Instrumentation

The use of inertial sensors for biomechanical analyses provide an accurate and portable method for analyzing segmental kinematics and has the advantage of being readily adaptable for use both on land and in water [26, 27]. Few researchers have used inertial sensors to assess underwater kinematics, however a recent study reported sagittal and frontal plane kinematics for the lower limbs and trunk during underwater gait [28]. The inertial sensors used in this study were waterproof and contained tri-axial accelerometers and gyroscopes (100 Hz) (Nanotrak, Catapult sports, Docklands, VIC). Each sensor has its own internal coordinate system and with the direction of segmental rotations differing both between left and right sides and between segments during the exercises (i.e. in the sagittal plane, the left thigh and right shank rotated in an anticlockwise direction during the ascending phase, whilst the left shank and right thigh rotated in a clockwise direction), the individual sensors differed in recording positive or negative values. Therefore, the data was adjusted so recordings from all sensors complied with a global coordinate system with the positive Y-axis directed anteriorly, the positive X-axis directed from left to right and the positive Z-axis pointing vertically. Four sensors were attached bilaterally to the participant’s lateral mid-thigh and shank, half-way between the proximal and distal joint centers and one sensor was positioned over the spinous process of the third thoracic vertebra. The allocation of the sensors was measured to be at equal distance from the proximal and distal joint centers for the lower body segments to ensure consistency, although researchers have highlighted that a considerable advantage of portable systems is that they are less sensitive to exact placement on the segments [26]. To measure squat depth, one additional sensor was attached to the sacrum, at equal distance from the posterior superior iliac spines. Before each exercise, a static calibration was performed with the participant standing still in an upright posture for ten seconds to establish 0^o^ orientations for the sensors and identify any offset in sensor allocations [28]. Each sensor was attached to the participant using 38mm rigid sports tape, and to avoid any intra-sensor bias, the same sensor was allocated to the same segment for all participants.

### Experimental protocol

Each participant attended two testing sessions; one land-based and one water-based, both with identical testing protocols and occurring within one week of each other. Following a self-selected warm up that included a few minutes of aerobic activity, stretches and between five and ten practice repetitions of each exercise for familiarization of the protocols and environments, the participants performed ten repetitions of the exercises; squat, SS, and SLS. All unilateral exercises were performed on both legs however, to avoid any bilateral asymmetries associated with leg dominance [29], the dominant leading leg data is presented. In order to capture each participants’ natural technique, and to ensure consistency between environments, no instructions were provided concerning foot positions or depth of the exercises [30]. Participants were instructed to maintain their elbows extended, palms facing down, arms straight and horizontal during all exercises, and during the SLS, the contralateral leg was flexed at the knee to between 70–90°, and kept behind the participant during the task. Participants performed all exercises to a tempo indicated by a metronome [31, 32] set at 100 beats per minute with four beats during the ascent and four beats during the descent, and were allowed between one and two minutes rest between the exercises. No randomization of the order of the exercises was used to allow the same task familiarization for each participant, and due to the natural sequencing progression of the movements. Also, as this was an inaugural study on kinematics the traditional approach is to use a homogeneous sample to begin with and future research should assess other populations. We based this on the current needs and demands of the local population to make the study relevant and practical.

The second testing session occurred at an outdoor pool complex and took place within one week of the first session. To ensure a consistent water depth and allow between-subject comparisons, participants performed the exercises on a platform of adjustable height, which was set so the water depth was level with the greater trochanter on each individual participant. The pool was of Olympic standard that was 1.35 meters deep without the platform, and had lane-ropes in place to reduce water turbulence, and the water temperature was maintained at 29.1°C±1.0 during the testing period.

### Data processing

**Fig 1:**
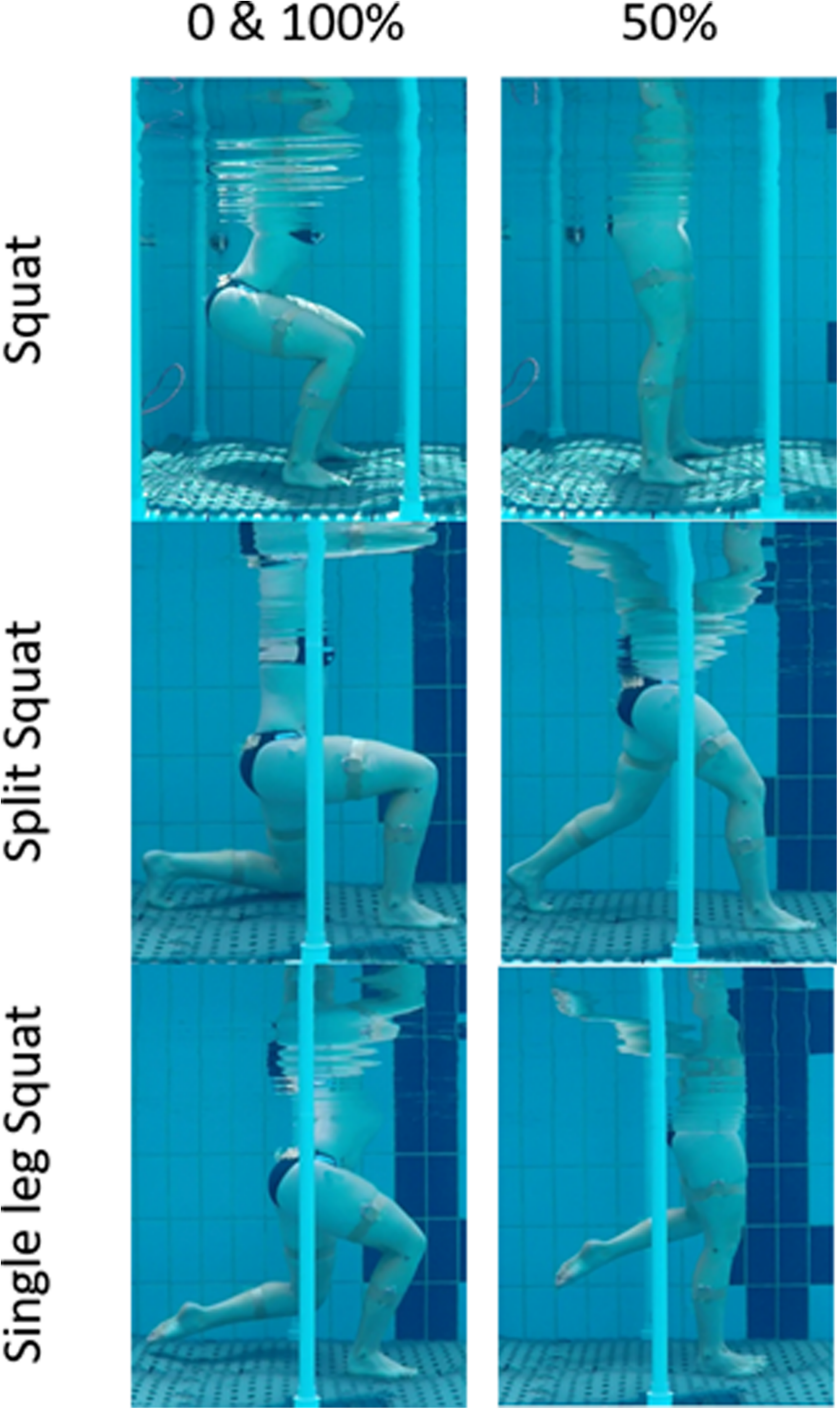
Exercise protocol. Participant performing the three exercises during immersion to highlight the top (0 and 100%) and bottom (50%) position of the three exercises.

The data from the inertial sensors was imported using Catapult Sprint (version 5.1; Catapult sports, Docklands, VIC), and the raw data from the gyroscopes (angular velocity in three spatial dimensions) was extracted into a comma-separated value (.csv) file. Each of the three gyroscope datasets were integrated and any gyroscopic drift was quantified with linear regression; the raw datasets were corrected for the drift and integrated again to yield non-drifting datasets of angular displacement as a function of time. The start and completion times of each repetition were identified from the minima and maxima of the dataset which had the largest amplitude of motion. This dataset was smoothed with a custom, variable-width, non-weighted box-smoothing algorithm, so that all true minima and maxima (peak angles) were correctly identified and false peaks due to noise were ignored, with a minimum amount of smoothing (excessive smoothing has potential to slightly “shift” maxima and minima). The individual repetitions in each of the three datasets were extracted, collated into sets, and processed. The data was processed so that the start of each cycle occurred at the bottom of the movement where the peak angle was most obvious, so for the analysis, the start of the movement (0%) represented the bottom of each task (the point of peak knee flexion), and subsequently, the top of the movement (point of peak knee extension) occurred around 50% (Fig 1). Further, the accelerometer data from the sacral sensor was used to determine its vertical displacement, which indicated movement depth [20].

### Data analysis

The data for each of the ten repetitions was time-normalized to 1000 data points and imported to Microsoft Excel 2016 (Microsoft Corporation, Redmond, WA) for comparison and analysis. As previous research has questioned the accuracy of transverse plane data recorded with inertial sensors [27, 28], only sagittal and frontal plane kinematics was analyzed in this study. All statistical analyses were performed with IBM SPSS software version 22 (IBM, New York, NY). Absolute values were used for all velocity data to allow comparisons between environments throughout both the ascending and descending phases, so it is portrayed as non-directional speed of movement. To identify differences between the phases of movement, time series data for displacements and speeds were divided into four phases; the early ascent (0–25%), late ascent (25–50%), early descent (50–75%) and late descent (75–100%). Kinematic variables of interest included the average angular displacement and speed for each phase, total range of motion, and peak velocities. The movement depths were tested for covariance, and all kinematic variables were tested for compliance with the assumptions of an analysis of covariance. Wherever the assumptions were met, an analysis of covariance determined significant differences between the environments, and elsewhere, a repeated measures one-way analysis of variance was used.

To allow comparison of environmental differences in displacements and movement speed throughout the tasks, the differences between the group mean waveforms ±95% confidence limits (95% CI) were plotted as a time series [33, 34] for the full movements (0–100%). The 95% CI was calculated using the critical *t*-value and degrees of freedom, and wherever it (shaded areas on figures) did not include zero, the environments were considered to have a significant effect on the variable. The mean differences were calculated as the land-based values less the aquatic-based values, thus a shaded area above zero indicated a trend of higher recorded values on land, and vice versa. variability of the individual waveforms was analyzed by calculating the coefficient of variance (CV), with additional calculations analyzing variability in pattern (CV_P_) and offset (CV_O_) [35] in both environments. The latter two techniques have been applied successfully to cyclical data and have been shown to be more sensitive to changes in movement patterns than the more traditional CV analysis techniques [35, 36].To portray the influence of the changed environment on variability, the differences between environments are presented as the land-based percentage less the pool-based percentage. Effect sizes were calculated and ranked using the method developed by Cohen [37], with scores d>0.2 considered small, d>0.5 moderate and d>0.8 considered large effect. The alpha level was set at p<0.05.

## Results

The analysis showed that immersion in water did not significantly affect the depth of the squat (land: 0.43±0.18 m, pool: 0.45±0.15 m, p = 0.700, d = 0.12) and SS (land: 0.34±0.06 m, pool: 0.38±0.09 m, p = 0.091, d = 0.53). However, the environment had a significant effect on the depth of the SLS (land: 0.22±0.09 m, water: 0.31±0.11 m, p = 0.006, d = 0.89).

**Fig 2:**
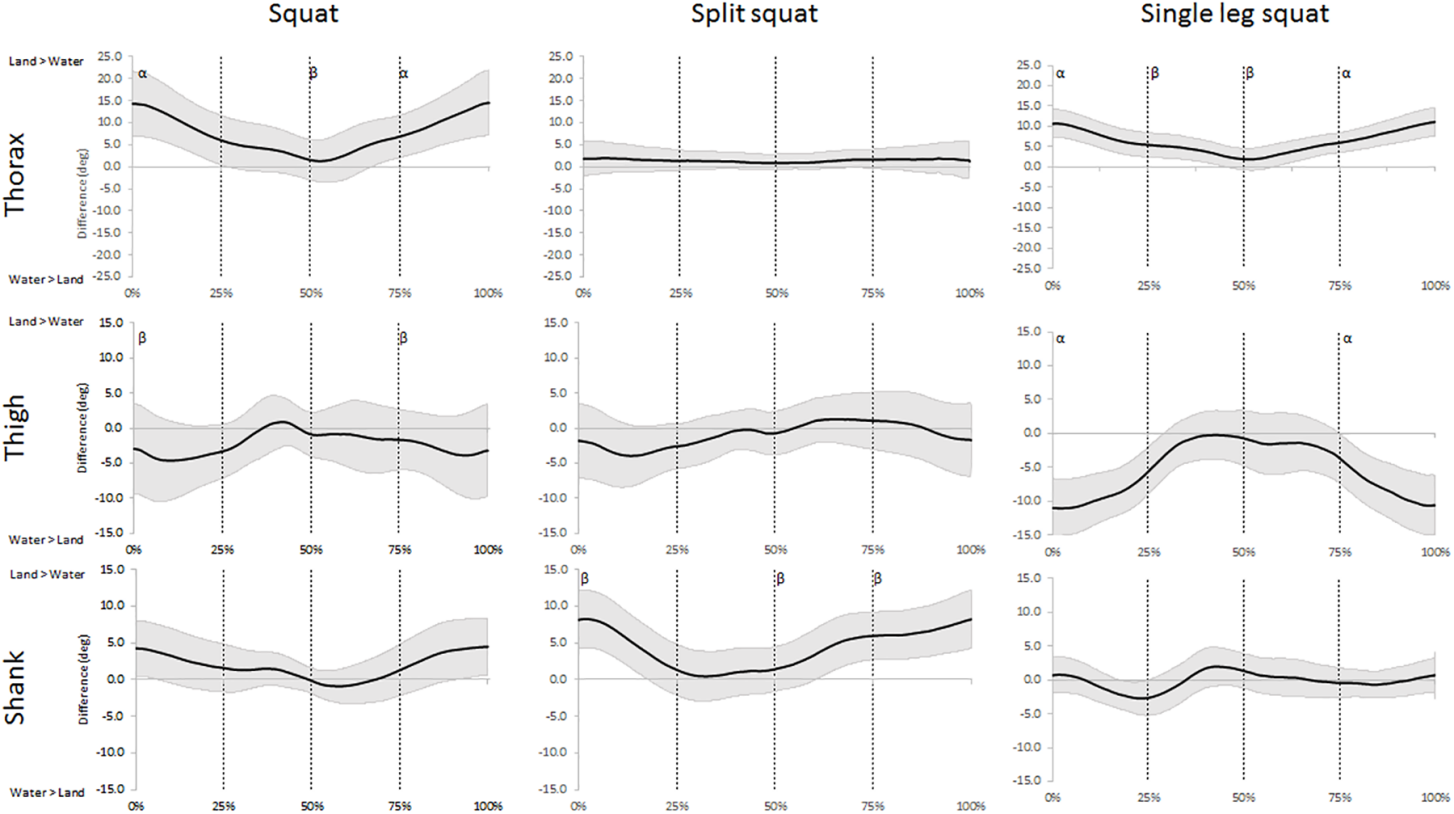
Waveforms of the mean difference (±95% CI) of sagittal plane displacements for the three segments between land- and aquatic-based squats during the movements. Differences between the group means (solid line) ±95% confidence limits (shaded area) for sagittal plane displacements for the thorax, thigh, and shank segments between land- and aquatic-based during the squat, split squat, and single leg squat throughout the movement. 95% confidence interval above zero indicates larger segmental inclination on land and vice versa. Vertical lines indicate the start and end of each phase; early ascent (0–25%), late ascent (25–50%), early descent (50–75%) and late descent (75–100%). ^α^ indicates a *large* environmental effect size at Cohen’s D >0.8, ^β^ indicates a *moderate* environmental effect size at Cohen’s D >0.5.

**Fig 3:**
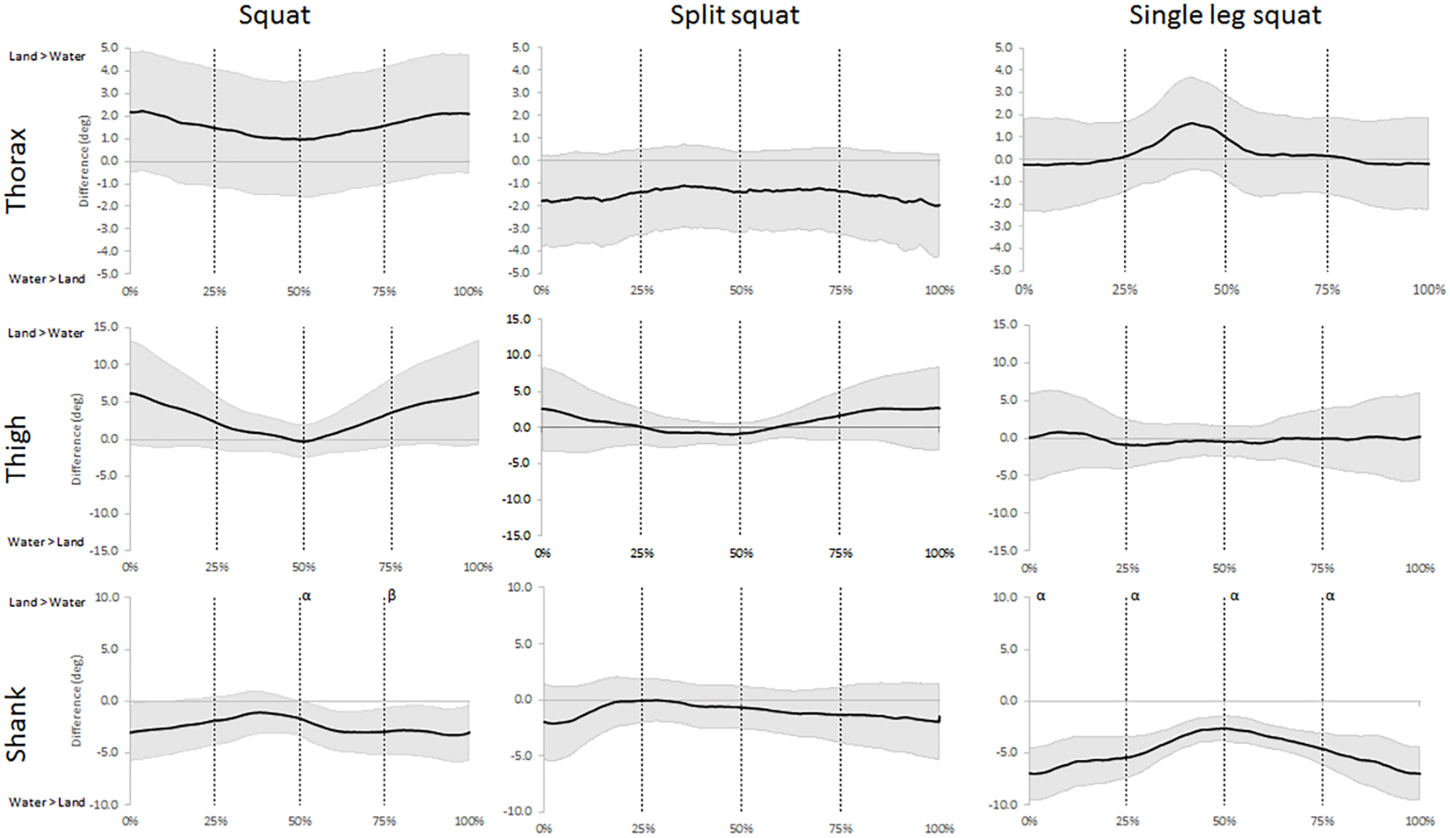
Waveforms of the mean difference (±95% CI) of frontal plane displacements for the three segments between land- and aquatic-based squats during the movements. Differences between the group means (solid line) ±95% confidence limits (shaded area) for the thorax, thigh, and shank segments between land- and aquatic-based during the squat, split squat, and single leg squat throughout the movement. 95% confidence interval above zero indicates larger segmental inclination on land and vice versa. Vertical lines indicate the start and end of each phase; early ascent (0–25%), late ascent (25–50%), early descent (50–75%) and late descent (75–100%). ^α^ indicates a *large* environmental effect size at Cohen’s D >0.8, ^β^ indicates a *moderate* environmental effect size at Cohen’s D >0.5.

**Fig 4:**
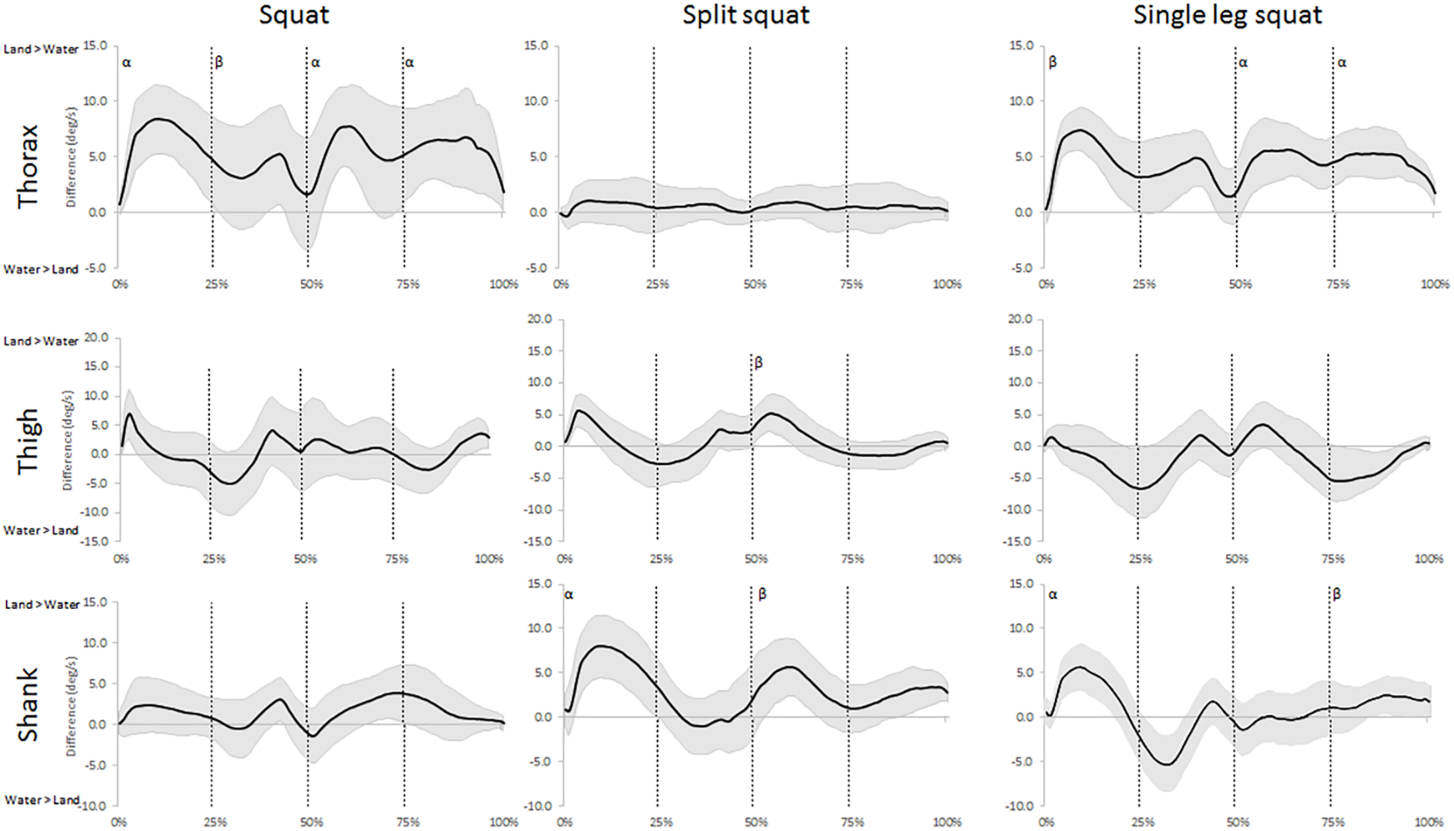
Waveforms of the mean difference (±95% CI) of sagittal plane movement speeds for the three segments between land- and aquatic-based squats during the movements. Differences between the group means (solid line) ±95% confidence limits (shaded area) for the thorax, thigh, and shank segments between land- and aquatic-based during the squat, split squat, and single leg squat throughout the movement. 95% confidence interval above zero indicates faster segmental speed on land and vice versa. Vertical lines indicate the start and end of each phase; early ascent (0–25%), late ascent (25–50%), early descent (50–75%) and late descent (75–100%). ^α^ indicates a *large* environmental effect size at Cohen’s D >0.8, ^β^ indicates a *moderate* environmental effect size at Cohen’s D >0.5.

**Fig 5:**
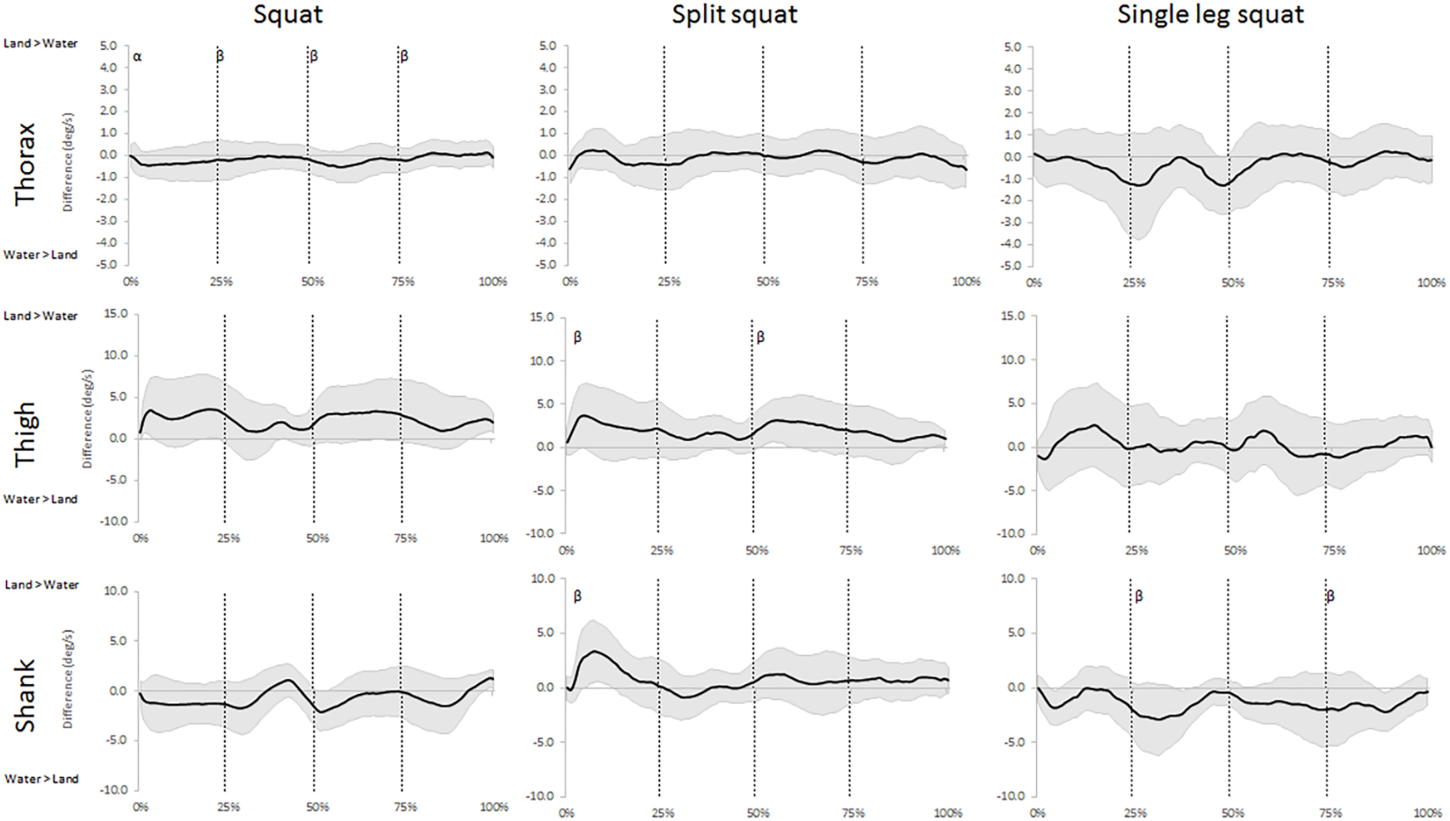
Waveforms of the mean difference (±95% CI) of frontal plane movement speeds for the three segments between land- and aquatic-based squats during the movements. Differences between the group means (solid line) ±95% confidence limits (shaded area) for thorax, thigh, and shank segments between land- and aquatic-based during the squat, split squat, and single leg squat throughout the movement. 95% confidence interval above zero indicates faster segmental speed on land and vice versa. Vertical lines indicate the start and end of each phase; early ascent (0–25%), late ascent (25–50%), early descent (50–75%) and late descent (75–100%). ^α^ indicates a *large* environmental effect size at Cohen’s D >0.8, ^β^ indicates a *moderate* environmental effect size at Cohen’s D >0.5.

The analysis of the angular displacement time series showed that water immersion had *moderate* and *large* effects on all segments in the sagittal plane during at least one exercise (Fig 2 and S1 Fig). Though, only the movements of the shank segment were effected in the frontal plane (Fig 3 and S2 Fig). *Moderate* and *large* effects were also observed in the movement speeds in the both planes of motion in all three exercises (Fig 4 and Fig 5). The waveforms also revealed differences in both orientation and speeds that differed between the phases when performing these exercises immersed in water.

The CV analysis showed several *moderate* and *large* significant effects on the segments movement variability in both planes of motion, with CV values often larger in the aquatic environment (Table 1). Only the shank segment portrayed more variability on land in the frontal plane during the SLS. The individual CV values for each segment in the two environments are provided in the supplementary material (S1 Table). The overall range of motion and peak velocities were also affected by the changed environment, with the data presented in the supplementary material (S2 Table, and S3 Table).

**Table 1:** Difference in movement variability (%) between land and water for the segments during the three exercises.

		Shank		Thigh		Thorax	
		X	Y	X	Y	X	Y
Squat	CV_P (%)_	-8.5	-14.0	-0.2	-15.6^β^	-9.2^β^	-57.8
	CV_O_ (%)	-7.7	-3.5	-2.5	-13.5	-5.6^β^	-29.0
	CV (%)	-5.1	-3.4	-1.2	-6.6	-7.7*^α^	-0.2
SS	CV_P (%)_	-11.7*^β^	-16.3^β^	-3.6	-27.8*^β^	-6.6	-25.3
	CV_O_ (%)	-0.7	1.6	-1.1	-21.7*^β^	-7.6	14.7
	CV (%)	-4.8	-15.0^β^	-2.2	-15.9	-22.1^β^	-4.8
SLS	CV_P (%)_	-3.7	1.8	-1.8	-60.8*^α^	-6.8	-10.1
	CV_O_ (%)	-13.6	30.4	-4.2	-15.5	-2.6	-30.6
	CV (%)	-7.4	57.1	-0.4	-18.4	-4.8	-68.1

CV–Coefficient of variance, CV_P_−Coefficient of variance for pattern, CV_O_−Coefficient of variance for offset.

Negative percentages indicate larger movement variability in the aquatic environment.

^*^ indicates significant difference between environments at P<0.05.

^α^ indicates *large* effect size at Cohen’s d >0.8.

^β^ indicates *moderate* effect size at Cohen’s d>0.5.

## Discussion

Our study shows that immersion in water alters squat, SS, and SLS trunk and lower body kinematics in young, healthy adults. These results support previous research on the influence of immersion in water on gait kinematics [9, 28]. To the best of our knowledge, this is the first study to use inertial sensors to compare the kinematics of squat variations on land and in water. A key finding is that water immersion to the greater trochanter does not limit the depths of squats and SS, and allows participants to maintain a range of movement similar to that they typically use on land. However, the aquatic environment does allow performers increased squat depth during the SLS.

During squats and SLS, the sagittal plane orientation of the thorax was particularly affected by the immersion protocols (Fig 2). These changes were indicative of a more vertically aligned trunk posture throughout the movements, being particularly apparent closer to the bottom position of both tasks (0–25% and 75–100% in Fig 2). This is a positive find as research highlights the important role that maintaining an upright trunk during squats and lifting tasks has in minimizing spinal compressive loads and shear forces [16, 38], and decreasing reliance on passive structures for support [39]. The more vertically aligned trunk posture in the aquatic environment is most likely an indication of the added support provided by the water coupled with the influence of the buoyant force acting up through the thorax. Further, the forward inclination of the trunk during squats performed on land is no doubt a strategy to maintain balance, and the participants might feel unstable if they attempt to employ a more vertical trunk posture, as it shifts their center of mass backwards [40]. Therefore, it appears the gravitational offloading and viscosity of the water reduces the performers’ reliance on their body position for stability and allows them to use a more upright trunk posture. Theoretically, the trunk posture employed in the aquatic environment during squats and SLS reduces spinal forces beyond what is already achieved with buoyancy, and provides a more stable movement with less reliance on the individuals’ balance skills. Our study assesses kinematic variables, so future research is needed to examine kinetic implications of water immersion during these exercises to provide additional understanding of the mechanical aspects of water-based exercise. However, it remains important for practitioners to understand the kinematic implications of water immersion to assist in the prescription of exercises. For example, researchers have highlighted increased forward trunk inclination during squats in older populations and individuals with lower back pain [41, 42], so the upright posture in the aquatic environment is likely beneficial for these populations. These results suggest that practitioners can employ the aquatic environment to improve squatting depth while simultaneously minimize spinal loads and improving trunk orientation. Further, although the analysis showed moderate and large effect sizes on the frontal plane speed of the trunk during the squat between the environments, these differences are probably too small to be of clinical importance.

The additional support in the aquatic environment is also evident in more vertically aligned shanks during the squats and SS, especially during the deeper phases of the tasks as the performer can ‘sit back’ in the movement without compromising balance. Again, this is a positive find as the upright shank positions are associated with reduced strain on the knees [43]. Contrary to the other exercises, the SLS had a slight, temporary increase in sagittal plane shank inclination in the aquatic environment during the ascending phase, which probably was associated with balance. On land, participants employ a forward trunk inclination to maintain the center of mass within the base of support, but the supportive and offloading properties of the water allow them to maintain vertical trunk posture and instead shift their entire body forward (i.e. increasing their shank inclination), without compromising balance. The buoyant force provided by the water would both reduce joint loading and offer lifting assistance during the ascending phase [22]. Combined, this means that participants probably were less limited by muscle strength and balance when they performed SLS in water and were thus able to squat deeper. Research have previously reported different muscle activation patterns between land and water and suggested that the offloading and reduced movement speeds dictated the muscular responses [44]. Future research is needed to assess muscle activity and kinetics during water-based squat tasks to determine neuromuscular responses to water immersion. Unsurprisingly, our examination revealed faster sagittal plane movement speeds for the segments in the environment with larger movement range (Fig 4). However, when the ranges are similar, the speeds appear highly individual and the environmental effects differ throughout the movement phases, particularly for the thigh and shank. Although, there seems to be some tendency for faster speeds in water during the late ascent, which could be explained by the buoyancy force adding to the muscular force providing an upthrust [45]. These preliminary findings could indicate that practitioners can employ the aquatic environment to train movements their clients might be unable to perform on land, likely as a part of early rehabilitation. A reduced restriction of strength and balance would allow clients to perform exercises such as SLS will full range earlier in the water than what is possible on land.

Our data also reveal more frontal plane movements of the shank in the aquatic environment during the SLS and the descending phase of the squat (Fig 3). While the frontal plane speeds of both lower body segments show similar trends to the sagittal plane, few differences are large enough to be of clinical interest (Fig 5). Nevertheless, lower body mediolateral alignment is an important consideration during squat performance as increased translation is linked to knee instability and injury [14]. Despite the aquatic environment often is considered unstable [46], it is possible that the properties of water can benefit balance through a few different features: First, the offloading reduce limitations by muscular strength for stability, second, slower movements provide increased time for postural corrections [5, 6], and third, it is possible that density and viscosity of the fluid can provide some support. The combination of these aspects could explain the increased frontal plane shank movements employed by our participants as they utilized the water for improving their balance. Previous research suggests that the aquatic environment reduces muscle activity of prime movers due to gravitational offloading [47], and similar trends are likely occurring in the stabilizing muscles, although further research is needed for confirmation. The practical implications of the increased frontal plane movements during water-based SLS require further examinations of whether it affects the leg muscle activity, and whether any changes are beneficial for rehabilitation.

Reduced reliance on muscle force for stability can also explain the increased movement variability in the aquatic environment. Increased movement variability indicates that the performer adapts to the constant movements of the surrounding water. Previous research suggests that injury and pain changes movement patterns by reducing movement variability [48], leaving the individual with decreased ability to adapt to surroundings and consequently, reduced functionality [24]. The increased movement variability during these squat exercises in the aquatic environment can potentially assist in restoring the adaptability in an injured population, further supporting its use in rehabilitation. Interestingly, during the SLS the shank portrays less variability in the frontal plane while in water but maintains a larger movement range. This could be linked to the strategy of using the vicious fluid for balance that we proposed earlier. Performers would not be able to apply this strategy on land under full gravitational loading as no additional support is provided by the air. It is also possible that the balance strategies employed on land are more variable than those applied in water, however future research should examine this further. Comparative research on movement variability in aquatic settings is lacking, thus preventing further comparisons and conclusions regarding its clinical significance.

One limitation of our study is that although our sensor allocation was thorough, there is a risk of slight discrepancies in sensor positions between testing sessions and participants. However, our method of landmark identification is the same as is used in practical settings and previous research and the risk of errors should be further reduced with the static capture [28]. Further, the sensors we used did not contain magnetometers, which potentially increased their susceptibility to internal drift [27], but the analysis compared only data recorded with the same sensor in the two environments, and any drift remaining after the filtering should be the same within each sensor. Additionally, we acknowledge that the greater variability in the water might be attributed to the participants performing the exercises in a novel environment. All participants were experienced in performing the exercises on land, but had not performed the exercises in water prior to the day of testing. It is possible that the inexperience of the participants increased their movement variability in the water. Future research should assess if habituation decreases the movement variability in the aquatic environment to further the research into this area. Further, researchers have shown kinematic differences between males and females during squatting tasks [49], and changing the depth of immersion can potentially also affect the kinematics of the exercises [46]. However, the small sample size of this study did not allow for analysis of differences between sexes and we limited our analysis to one depth however, we highlight that future research should assess if water immersion affects the kinematics differently between sexes, and quantify implications of different water depths on kinematics.

## Conclusion

This study reveals several kinematic differences between land and water when healthy adults perform bodyweight squats, SS and SLS. Our data shows that immersion in water to the greater trochanter does not limit the overall movement range or depth during the squat and SS, while it allows performers to achieve greater depth during the SLS. The aquatic environment encourages more vertically aligned trunk and shank segments with an overall smaller range of motion, which consequently decreases the speed of the segments. We also observe increased motions in the frontal plane during water-based SLS, and that all three exercises show increased movement variability in water. This study also highlights the need for further research into the applications of water-based squatting tasks in order to provide practitioners with a more comprehensive understanding of movement mechanics in water. Combined, the findings of our study highlight the suitability of aquatic-based squats, SS and SLS for lower body rehabilitation as water immersion emphasizes improved technique without changing the overall movement pattern.

## Supporting information

S1 Fig Displacement waveforms of sagittal plane movements for the three segments between land- and aquatic-based squats. **Average sagittal plane displacement on l**and (solid line) ±95% confidence limits (green area), and in water (dashed line) ±95% confidence limits (blue area) for thorax, thigh, and shank segments during the squat, split squat, and single leg squat. Vertical lines indicate the start and end of each phase; early ascent (0–25%), late ascent (25–50%), early descent (50–75%) and late descent (75–100%).
(DOCX)

S2 Fig Displacement waveforms of frontal plane movements for the three segments between land- and aquatic-based squats. **Average frontal plane displacement on l**and (solid line) ±95% confidence limits (green area), and in water (dashed line) ±95% confidence limits (blue area) for thorax, thigh, and shank segments during the squat, split squat, and single leg squat. Vertical lines indicate the start and end of each phase; early ascent (0–25%), late ascent (25–50%), early descent (50–75%) and late descent (75–100%). Positive values indicate valgus movements at the thigh and shank.
(DOCX)

S1 Table Movement variability (%) for the three segments in the environments. CV–Coefficient of variance, CV_p_−Coefficient of variance for pattern, CV_O_−Coefficient of variance for offset
(DOCX)

S2 Table Mean (SD) range of motion and the movement variability (%) between the two environments during the concentric phase of the movement. CV, coefficient of variability Positive percentages indicate larger movement variability in the aquatic environment, and negative percentages indicates larger movement variability on land.
* indicates significant difference between environments at P<0.05.
α –indicates *large* effect size at Cohen’s d >0.8.
β –indicates *moderate* effect size at Cohen’s d>0.5.
(DOCX)

S3 Table Mean (SD) peak velocity between the two environments during the concentric phase of the movement. X, Extension Y, Abduction Z, External rotation.
CV, coefficient of variability.
Positive percentages indicate larger movement variability in the aquatic environment, and negative percentages indicates larger movement variability on land.
* indicates significant difference between environments at P<0.05.
α –indicates *large* effect size at Cohen’s d >0.8. β –indicates *moderate* effect size at Cohen’s d>0.5.
(DOCX)
