# Supplementary material for: Quantifying kinematic differences between land and water during squats, split squats, and single-leg squats in a healthy population
Source: PLoS One. 2017 Aug 2;12(8):e0182320. doi: 10.1371/journal.pone.0182320 (PMC5540590; doi:10.1371/journal.pone.0182320)
Supplement: S2 Fig — Average frontal plane displacement on land (solid line) ±95% confidence limits (green area), and in water (dashed line) ±95% confidence limits (blue area) for thorax, thigh, and shank segments during the squat, split squat, and single leg squat. Vertical lines indicate the start and end of each phase; early ascent (0–25%), late ascent (25–50%), early descent (50–75%) and late descent (75–100%). Positive values indicate valgus movements at the thigh and shank. (DOCX) [file pone.0182320.s002.docx]

**S2 Fig Displacement waveforms of frontal plane movements for the three segments between land- and aquatic-based squats. Average frontal plane displacement on l**and (solid line) ±95% confidence limits (green area), and in water (dashed line) ±95% confidence limits (blue area) for thorax, thigh, and shank segments during the **squat, split squat, and single leg squat**. Vertical lines indicate the start and end of each phase; early ascent (0-25%), late ascent (25-50%), early descent (50-75%) and late descent (75-100%). Positive values indicate valgus movements at the thigh and shank.
