# Supplementary material for: Quantifying kinematic differences between land and water during squats, split squats, and single-leg squats in a healthy population
Source: PLoS One. 2017 Aug 2;12(8):e0182320. doi: 10.1371/journal.pone.0182320 (PMC5540590; doi:10.1371/journal.pone.0182320)
Supplement: S1 Table — CV–Coefficient of variance, CVp−Coefficient of variance for pattern, CVO−Coefficient of variance for offset (DOCX) [file pone.0182320.s003.docx]

**S1 Table. Movement variability (%) for the three segments in the environments**

|  | Shank | | | | Thigh | | | Thorax | | |
| --- | --- | --- | --- | --- | --- | --- | --- | --- | --- | --- |
|  | CV_P_ (%) | | CV_O_ (%) | CV (%) | CV_P_ (%) | CV_O_ (%) | CV (%) | CV_P_ (%) | CV_O_ (%) | CV (%) |
| Squat: Land | | |  |  |  |  |  |  |  |  |
| X | 32.1 | | 14.1 | 17.9 | 17.3 | 9.6 | 10.8 | 29.2 | 16.4 | 17.5 |
| Y | 42.2 | | 31.8 | 31.5 | 27.9 | 28.5 | 21.5 | 159.9 | 179.6 | 43.8 |
| Pool | |  |  |  |  |  |  |  |  |  |
| X | 40.6 | | 21.7 | 23.0 | 17.5 | 12.1 | 12.0 | 38.4 | 22.0 | 25.2 |
| Y | 56.3 | | 35.4 | 35.0 | 43.6 | 42.0 | 28.1 | 217.6 | 208.6 | 44.0 |
| Split Squat: Land | | | |  |  |  |  |  |  |  |
| X | 27.3 | | 17.8 | 18.3 | 16.5 | 9.2 | 13.1 | 138.9 | 144.4 | 52.1 |
| Y | 40.8 | | 36.9 | 30.1 | 32.5 | 26.1 | 30.3 | 286.7 | 301.4 | 65.3 |
| Pool | |  |  |  |  |  |  |  |  |  |
| X | 39.0 | | 18.5 | 23.1 | 20.2 | 10.2 | 15.3 | 145.5 | 152.1 | 74.2 |
| Y | 57.1 | | 35.2 | 45.1 | 60.2 | 47.8 | 46.3 | 311.9 | 286.7 | 70.1 |
| Single leg Squat: Land | | | |  |  |  |  |  |  |  |
| X | 38.1 | | 21.2 | 24.8 | 28.1 | 16.4 | 19.4 | 32.6 | 23.7 | 26.5 |
| Y | 100.2 | | 106.9 | 110.9 | 48.0 | 71.0 | 52.0 | 226.8 | 151.2 | 42.8 |
| Pool | |  |  |  |  |  |  |  |  |  |
| X | 41.8 | | 34.8 | 32.2 | 29.9 | 20.6 | 19.8 | 39.4 | 26.3 | 31.4 |
| Y | 98.4 | | 76.5 | 53.8 | 108.7 | 86.5 | 70.4 | 236.9 | 181.8 | 110.8 |

CV – Coefficient of variance, CV_p_ – Coefficient of variance for pattern, CV_O_ – Coefficient of variance for offset
