# Supplementary material for: Quantifying kinematic differences between land and water during squats, split squats, and single-leg squats in a healthy population
Source: PLoS One. 2017 Aug 2;12(8):e0182320. doi: 10.1371/journal.pone.0182320 (PMC5540590; doi:10.1371/journal.pone.0182320)
Supplement: S2 Table — CV, coefficient of variability Positive percentages indicate larger movement variability in the aquatic environment, and negative percentages indicates larger movement variability on land. * indicates significant difference between environments at P<0.05. α –indicates large effect size at Cohen’s d >0.8. β –indicates moderate effect size at Cohen’s d>0.5. (DOCX) [file pone.0182320.s004.docx]

**S2 Table. Mean (SD) range of motion and the movement variability (%) between the two environments during the concentric phase of the movement.**

|  | Shank | | Thigh | | Thorax | |
| --- | --- | --- | --- | --- | --- | --- |
|  | X | Y | X | Y | X | Y |
| Squat |  |  |  |  |  |  |
| Land (°) | 25.7 ± 8.3 | 10.7 ± 6.1 | 68.7 ± 10.5 | 19.1 ± 11.9 | 36.3 ± 13.1 | 2.0 ± 0.7 |
| Pool (°) | 22.1 ± 6.5 | 12.3 ± 6.1 | 70.4 ± 14.2 | 15.5 ± 12.1 | 24.1 ± 8.9*^α^ | 2.2 ± 1.4 |
| Split Squat |  |  |  | |  |  |
| Land (°) | 31.8 ± 8.7 | 11.4 ± 6.6 | 49.5 ± 8.8 | 16.7 ± 10.2 | 7.9 ± 6.5 | 2.0 ± 1.3 |
| Pool (°) | 25.0 ± 8.8^β^ | 10.2 ± 5.3 | 50.7 ± 13.2 | 12.5 ± 9.2 | 4.0 ± 7.1^β^ | 2.6 ± 2.5 |
| Single leg squat |  |  |  | |  |  |
| Land (°) | 24.7 ± 7.2 | 7.2 ± 4.8 | 41.6 ± 9.3 | 11.0 ± 5.9 | 31.1 ± 12.4 | 4.3 ± 2.2 |
| Pool (°) | 25.3 ± 6.2 | 10.9 ± 6.0^β^ | 52.5 ± 12.3*^α^ | 12.1 ± 7.1 | 22.6 ± 10.0*^β^ | 4.3 ± 2.4 |

CV, coefficient of variability
Positive percentages indicate larger movement variability in the aquatic environment, and negative percentages indicates larger movement variability on land
* indicates significant difference between environments at P<0.05
α – indicates *large* effect size at Cohen’s d >0.8
β – indicates *moderate* effect size at Cohen’s d>0.5
