# Supplementary material for: Quantifying kinematic differences between land and water during squats, split squats, and single-leg squats in a healthy population
Source: PLoS One. 2017 Aug 2;12(8):e0182320. doi: 10.1371/journal.pone.0182320 (PMC5540590; doi:10.1371/journal.pone.0182320)
Supplement: S3 Table — X, Extension Y, Abduction Z, External rotation. CV, coefficient of variability. Positive percentages indicate larger movement variability in the aquatic environment, and negative percentages indicates larger movement variability on land. * indicates significant difference between environments at P<0.05. α –indicates large effect size at Cohen’s d >0.8. β –indicates moderate effect size at Cohen’s d>0.5. (DOCX) [file pone.0182320.s005.docx]

**S3 Table Mean (SD) peak velocity between the two environments during the concentric phase of the movement.**

|  | Shank | | Thigh | | Thorax | |
| --- | --- | --- | --- | --- | --- | --- |
|  | X | Y | X | Y | X | Y |
| Squat |  |  |  | |  |  |
| Land (°∙s^-1^) | 19.5 ± 8.1 | 8.8 ± 4.3 | 42.9 ± 8.5 | 13.7 ± 8.9 | 23.1 ± 7.2 | 2.1 ± 0.9 |
| Pool (°∙s^-1^) | 20.2 ± 8.3 | 12.3 ± 8.4^β^ | 48.7 ± 18.6 | 14.5 ± 12.8 | 18.9 ± 10.0 | 2.7 ± 2.0 |
| Split squat |  |  |  | |  |  |
| Land (°∙s^-1^) | 24.1 ± 8.9 | 10.3 ± 5.5 | 33.2 ± 9.2 | 14.6 ± 10.9 | 6.6 ± 5.2 | 2.8 ± 2.2 |
| Pool (°∙s^-1^) | 20.0 ± 7.3^β^ | 10.1 ± 4.3 | 23.9 ± 8.9 | 10.9 ± 5.2 | 6.7 ± 4.7 | 3.1 ± 2.7 |
| Single-leg squat |  |  |  | |  |  |
| Land (°∙s^-1^) | 7.9 ± 3.8 | 10.3 ± 5.5 | 30.7 ± 9.7 | 14.6 ± 9.8 | 21.6 ± 8.1 | 2.8 ± 2.2 |
| Pool (°∙s^-1^) | 11.4 ± 6.4^β^ | 10.4 ± 4.3 | 35.2 ± 13.3 | 10.9 ± 5.2 | 17.4 ± 7.0^β^ | 3.1 ± 2.7 |

X, Extension Y, Abduction Z, External rotation
CV, coefficient of variability
Positive percentages indicate larger movement variability in the aquatic environment, and negative percentages indicates larger movement variability on land
* indicates significant difference between environments at P<0.05
α – indicates *large* effect size at Cohen’s d >0.8
β – indicates *moderate* effect size at Cohen’s d>0.5
